# Synthesis, characterization, synergistic inhibition, and biological evaluation of novel Schiff base on 304 stainless steel in acid solution

**DOI:** 10.1038/s41598-023-51044-w

**Published:** 2024-01-04

**Authors:** Shimaa Hosny, Aliaa Abdelfatah, Ghalia A. Gaber

**Affiliations:** 1 Chemistry Department, Faculty of Science, New Valley University, El-Kharga, 72511 Egypt; 2 Mining, Petroleum and Metallurgical Engineering Department, Faculty of Engineering, Cairo University, Cairo, Egypt; 3 Department of Chemistry, Faculty of Science (Girls), Al-Azhar University, Yousef Abbas Str., P.O. Box: 11754, Nasr City, Cairo, Egypt

**Keywords:** Inorganic chemistry, Chemical bonding

## Abstract

A novel Schiff base [4-(morpholin-4-yl) benzylidenyl]thiosemicarbazide (MBT) was created by reaction condensation. The molecules of the products were verified by IR, ^1^HNMR, MS, and elemental techniques. The synergistic effect of KI with novel MBT on 304 stainless steel (SS) in acidic has been investigated experimentally and theoretically using DFT. The findings demonstrate that restriction efficacy on 304 SS improved with rising inhibitor concentrations, and this benefit was attributed to synergy when KI was injected. From EIS results, IE % increased with a higher concentration of MBT only and MBT + KI (from 100 to 600 ppm). MBT maximum IE % was 84.98%, at 600 ppm. MBT + KI, due to the I^−^ ions synergistic effect, showed an IE% of about 95.48%, at 600 ppm. The adsorptions of MBT and MBT + KI on the surfaces of 304 SS are strongly fitted Langmuir adsorption isotherms. Thermodynamic parameters (K_ads_, ΔG^0^_ads_) were utilized. According to polarization findings, MBT behaves as a mixed-category antagonist. The Schiff base MBT was screened for its in vitro antimicrobial activities against some strains of bacteria and fungi. The result revealed that MBT proved to be an excellent candidate as a fungal agent being able to inhibit Aspergillus flavus.

## Introduction

One of the main industrial issues is corrosion, which causes damage to many petroleum installations, including reservoirs, distillation towers, and oil pipelines. Industries have implemented several techniques, including galvanizing, electroplating, and utilizing inhibitors, to reduce losses. However, due to its simplicity and affordability, using an inhibitor is thought to be the best method among them^1–5^. As corrosion inhibitors, a variety of inorganic and organic, those with aromatic rings, N, S, and O atoms, have been researched and used more effectively, more affordable, environmentally benign, and non-toxic^6–8^. The best approach to stop the corrosion of 304 SS and GS in acidic is to utilize specific morpholinobenzyldine compounds^9^. Due to the π-orbital with the surface, compounds have high inhibitive characteristics^10^. Many compounds of Schiff bases that have an azomethine bond function as potent corrosion inhibitors^11^. The effectiveness of a compound can occasionally be increased by including additional chemicals that work in synergism. Numerous studies on the synergistic effect have been conducted and are being reviewed. The order of Cl, Br, and I has been seen to increase the synergistic action of the halides. Iodide exhibits the largest synergistic impact due to its size and polarizability^12^. Many organic compounds' corrosion on mild steel was studied. The potency of the hydrazide derivatives as corrosion inhibitors in acidic has been thoroughly researched^9^. To determine whether it is possible to relate chemical structures to inhibitory effects, the researchers also looked into quantum chemistry^13^. Recent advancements in prevention and control center on finding new organic molecules by improving upon the existing ones. The anticancer efficacy of a few carbohydrazide derivatives with furan units was synthesized, characterized, and reported. However, due to the molecular architectures and the presence of heteroatoms, the molecules' capacity to prevent metal corrosion was further studied using theoretical principles such as density functional theory (DFT) techniques^14–19^. Therefore, we report herein the synthesis of a novel Schiff base, of [4-(morpholin-4-yl) benzylidenyl]thiosemicarbazide(MBT) by collection of 4-morpholinobenzaldehyde with thiosemicarbazide in ethanol. The synthesized Schiff base has been characterized based on their elemental analysis, FT-IR, ^1^HNMR, and mass spectral data. The impact of Schiff base on the corrosion and synergist of it with KI for 304 stainless steel (SS) in 1 M HCl was studied by gravimetric method, potentiodynamic polarization, and electrochemical impedance spectroscopy (EIS) measurements. Furthermore, the in vitro antimicrobial synthesized Schiff base was also evaluated against some strains of bacteria and fungi. The results were compared with quantum chemical data.

## Experimental details

### Spectral studies

A Perkin-Elmer elemental analysis (240c) was used to evaluate the CHNS contents. The melting point of the synthesized Schiff base was determined using the Gallenkamp equipment. FTIR spectra were acquired on a Shimadzu spectrophotometer using a KBr pellet in the (4000–400 cm^−1^) range. On a BRUKER 400 MHz, 1HNMR spectra in DMSO-d6 were acquired. Mass spectra were obtained using a (MS-5988 GC–MS) set at 70 eV. Thermogravimetric analyses were carried out on a Shimadzu DTG-60H in an inert environment at 10 °C/min.

### Synthesis of MBT

**Figure 1:**
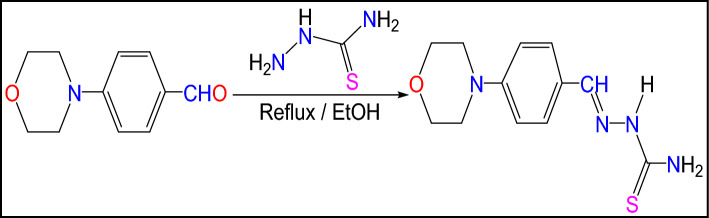
Synthesis of studied inhibitor (MBT).

A novel Schiff base, of [4-(morpholin-4-yl)benzylidenyl]thiosemi-carbazide (MBT) was synthesized by condensation of 4-morpholinobenzaldehyde (0.01 Mol dm^–3^) with thiosemicarbazide (0.01 mol dm^–3^) in 50 ml ethanol. The progress of the reaction was monitored by TLC. The synthetic route is outlined in (Fig. 1). The solid product formed was collected by filtration and recrystallized from ethanol as red crystals; yielding 85%; mp. 270 °C.

Yield: 85%; M.P. 270 oC. UV–Vis spectroscopy; the bands at λ_max_ (386, 445, 468, and 539 nm) are attributed to (π–π*) transition of the aromatic ring, (n–π*) transition of imine (C=N), (n–π*) transition of the phenolic group and charge-transfer (CT), respectively.

### Electrode assembly

The WE 304 stainless steel (SS) has compositions of 0.64% Si, 15% Cr, 5% Ni, 2% Mn, 0.02% S, 0.05% C, 0.043% P, and balance Fe. Uniform surfaces of 304 SS specimens were prepared with a grinding machine using emery paper of different grades (220, 400, 800, and 1000).

### Corrosive medium

The acidic solution was prepared by the dilution of A.R grade 37% of HCl with deionized H_2_O. The concentrations of Schiff base MBT were chosen as (100, 200, 400, and 600 ppm). By adding 0.1 M KI, the synergist of iodide ions was investigated.

### Corrosion studies

#### Gravimetric method

The gravimetric approach is the simplest way to evaluate corrosion losses. The samples are first properly cleaned, after which they are polished with different grades of SiC, washed in ethanol, rinsed in distilled H_2_O, and then allowed to dry. Following these stages, the specimens were precisely weighed before being submerged in 50 mL of 1 M HCl in both the presence and absence of various inhibitor doses (100, 200, 400, and 600 ppm). At a temperature of 25 °C, weight loss measurements were collected over 5 days. The specimens were removed from the test solution, completely cleaned of corrosion products, and then allowed to dry before being precisely reweighed^9^. Equation 1^20^ can be used to get the average weight reduction in grams:

1 $$\Delta {\text{W}} = W_{i} - W_{f} { }$$

where W_i_ = initial weight; W_f_ = final weight; ∆W = weight loss. The CR in mm/y is calculated in Eq. 2:

2 $${\text{CR}}\left( {{\text{mm}}/{\text{y}}} \right) = \frac{{\Delta {\text{W}} \times {\text{K}}}}{{{\text{A}} \times {\text{T}} \times {\text{D}}}}$$

where: K = 8.76 × 10^4^, T = time in h, A = area in cm^2^, ∆W = weight-loss in grams, and D = density in g/cm^3^. The inhibition efficiency (IE) and surface coverage (θ) are determined by Eqs. (3) and (4), respectively.

3 $${\text{IE \% }} = \frac{{W_{0} - W_{corr} }}{{W_{0} }} \times { }100$$

where W_corr_ and W_o_ are the loss in the presence and absence of inhibitor, separately^11^.

4 $${\text{Surface coverage }}\left( {\uptheta } \right) = \frac{{{\text{IE}}}}{100}$$

#### PDP method

At a scan rate of 2 mV/s, polarization was produced in the electrode potential range of − 700 to + 700 mV_Ag/AgCl_. To calculate current densities (I_corr_), Tafel polarization analysis of anodic and cathodic curves was performed. Using Eq. 5, the inhibitive efficiency (IE%) was computed.

5 $${\text{IE \% }} = 1 - \frac{{CR_{inh} }}{{{\text{CR}}}}{\text{ x }}100$$

where CR and CR_inh_ are the corrosion rates in the absence and presence of inhibitor, respectively^21^.

#### EIS method

The impedance was done directly after keeping the electrode for 30 min until a steady-state open circuit potential (OCP) was reached. Corrosion inhibition measurements comprising EIS as well as PDP curves were completed in a three-electrode cell assembly using a VoltaMaster PGZ 301 potentiostat/galvanostat connected to a laptop. In this cell, the reference electrode, counter electrode, and working electrode are saturated calomel electrodes (SCE), platinum sheet electrode, and 304 SS, respectively. Because Schiff base compounds adsorbed to the 304 SS takes a certain amount of time, the 304 SS samples were dipped in 1 M HCl having diverse doses of Schiff base compounds for 30 min. After that, an almost stable state could be obtained for the open circuit potential (OCP). NOVA 1.11.2 software was used to fit the EIS curves to assume the impedance parameters such as electrolyte resistance (R _s_), and charge transfer (R_ct_).

Utilizing AC signals at OCP, the electrochemical impedance of 304 SS was performed in the frequency range of 100 kHz to 100 mHz with an amplitude of 10 mV peak-to-peak. Using charge transfer resistance values from Eq. 6^22^, the IE% and θ were calculated.

6 $$IE \% = \theta \times 100 = 1 - \frac{Rct}{{Rct_{inh} }} \times 100$$

where Rct and Rct_(inh)_ are the charge transfer in the absence and presence of an inhibitor, respectively.

### Computational details

The Schiff base ligand was optimized with the Gauss View 09 program B3LYP functional with 6-311++G basic set^23^.

### Biological activity

The prepared Schiff base ligand were screened against *Bacillus cereus (G* + *ve), E. coli (G−ve), Micrococcus luteus (G* + *ve), Pseudomonas aeruginosa (G−ve), Serratia marcescens (G−ve), Staphylococcus aureus (G* + *ve) bacteria and Aspergillus flavus, candida albicans, Fusarium oxysporum, Geotrichum candidum, Scopulariopsis brevicaulis and Trichophyton rubrum fungi*.

## Results and discussion

### Characterization of inhibitor

#### Analytical data

The data from elemental analysis of the newly synthesized MBT ligand agreed with the assumed molecular formulae, Table S1. This demonstrates the Schiff base ligand's purity**.**

#### ^1^H-NMR interpretation

The ^1^H NMR of MBT in DMSO-d6 revealed the appearance of azomethine proton at δ: 7.70 ppm^24^. The NH proton signal was observed at δ: 8.10 ppm^25^. The signal at δ: 7.50 ppm for NH_2_ proton. In addition, the presence of morpholine and aromatic system at δ: 3.30 and 6.90 ppm^25^, respectively, indicates the purity of the prepared ligand (Fig. S1).

#### Infrared spectral of the Schiff base ligand (MBT)

The MBT ligand spectrum displayed absorption bands at 3174, 3340, and 758 cm-1, which are, respectively, attributed to (NH), (NH_2_), and (C=S) groups. The imine signal of the ligand (C=N) at 1623 cm^−1^ reveals the production of the MBT ligand. (Fig. S2).

#### Mass spectral data

**Figure 2:**
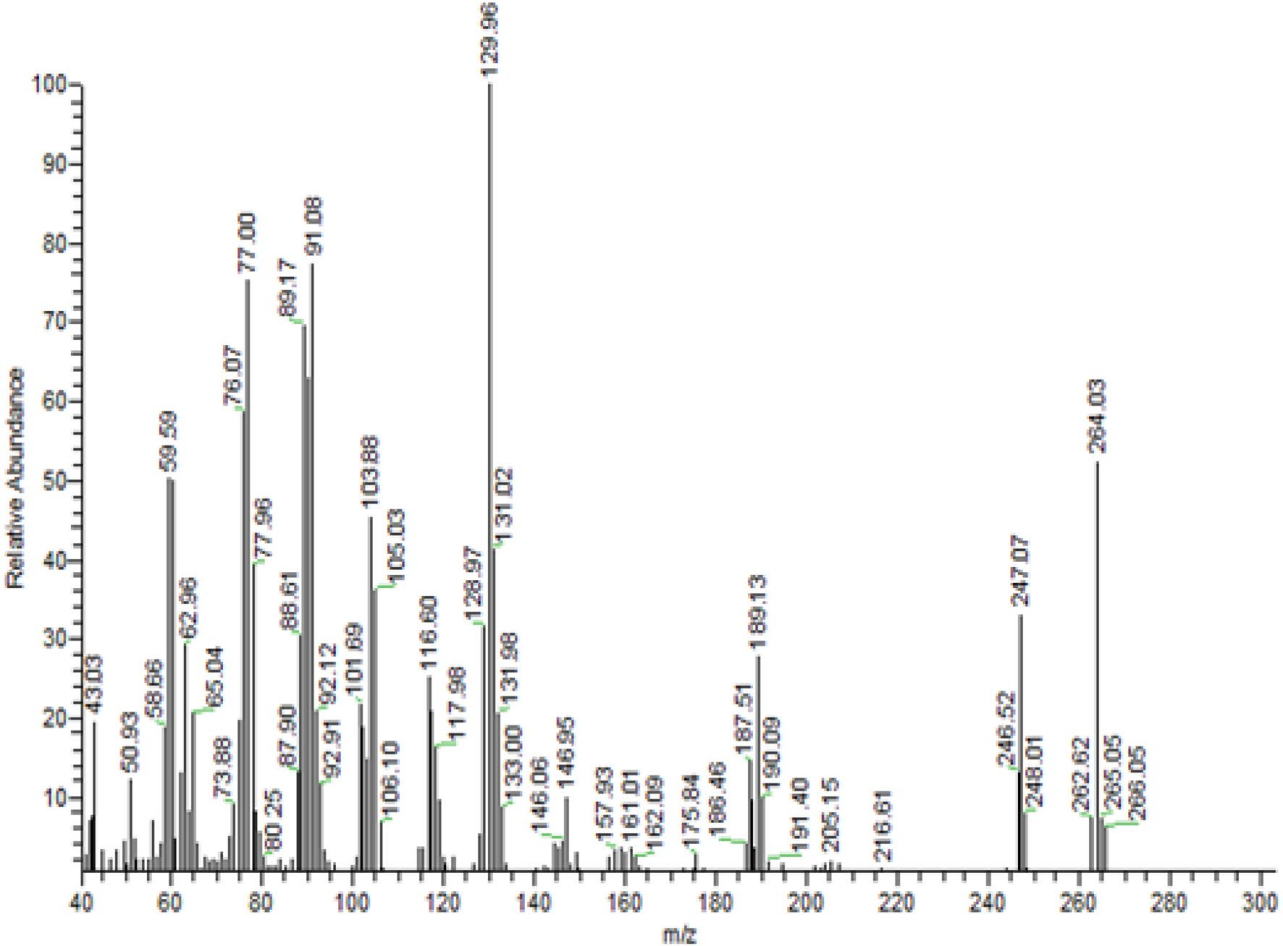
ESI–MS of the MBT Schiff base ligand.

**Figure 3:**
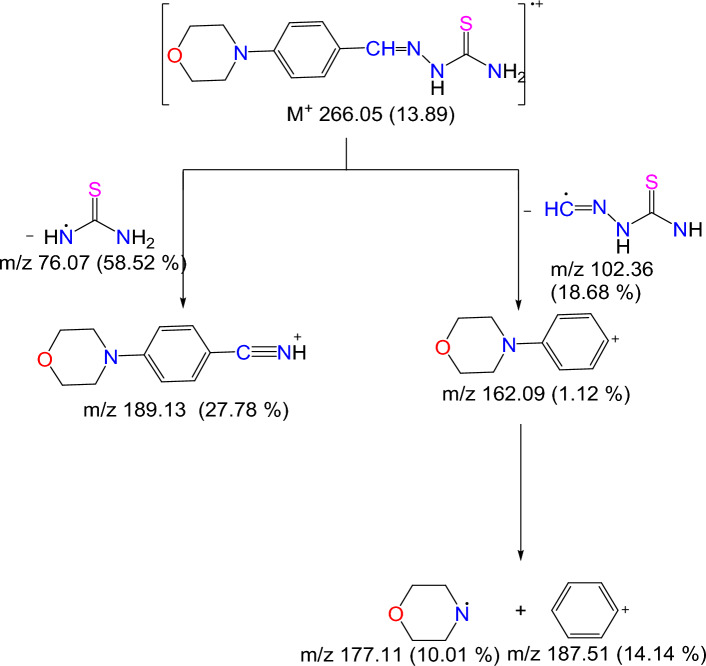
The fragmentation pathway of MBT.

The MS of MBT revealed a molecular ion peak (*m/z*) at *m/z*: 266.05, which corresponds to the predicted molecular weight (Fig. 2). The MBT fragmentation route is distinguished by the presence of molecular ion peaks at *m/z*: 189.13 (27.78%) for [M-C_11_H_13_ON_2_]^+^, at *m/z*: 187.51 (14.14%) for [M-C_6_H_4_]^+^ and base peak at *m/z*: 129.96 (100%), (Fig. 3).

#### Thermal analysis of MBT Schiff base ligand

The TG of MBT (Fig. S3) shows that completely decomposed with weight losses in two steps. The first step amounted to (calc. 38.5%, found 38.6%) at the temperature range between 116 and 428 °C, this can reveal the release of 2-methylene hydrazinecarbothioamide radical (^·^C_2_H_4_N_3_S). The second mass loss is commensurate with the release of a 4-phenylmorpholine radical (calc. 61.3%, found 61.4%) at the temperature range between 428 and 691 °C (Fig. S4).

### Corrosion studies

#### Gravimetric method

**Figure 4:**
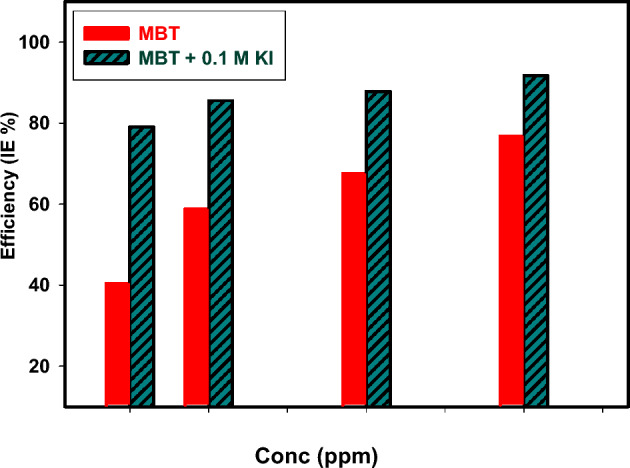
Correlation IE% of MBT and MBT + 0.1 M KI at 120 h.

At 120 h, the weight loss of 304 SS in 1 M HCl at 25 °C with varying doses of inhibitor MBT and inhibitor + KI was assessed. Table 1 lists the corrosion rates, inhibition effectiveness, and surface coverage for 304 SS specimens. It is evident that as the inhibitor concentration increased from 100 to 600 ppm, the corrosion rate significantly decreased. Because the inhibitor molecules' surface is covered on the metal by adsorption. The synergistic impact of halide ions caused the corrosion rate to decrease with the addition of KI. The correlation of IE % against concentration of MBT and MBT + 0.1 M KI is represented in Fig. 4. As extra concentration causes the IE% to climb from 40.41 to 76.84% in the case of MBT alone, but increased from 79.01% to high of 91.79% in the presence of MBT + 0.1 M KI, it can be concluded that the addition of KI significantly strengthened the synergistic effectiveness of MBT inhibition. By conducting an intensified corrosion blockage of the active sites on the surface exposed to the media, the molecule adsorption will be gradually improved^26^. For all examined concentrations, it was discovered that the combination of inhibitor and KI has better inhibitory efficiency when compared to the values of inhibitor without KI. This work firmly demonstrates the part played by the Schiff base azomethine linkage (C=N), which actively contributes to the inhibitory mechanism.

**Table 1:** Corrosion rate, standard deviation (S.D), surface coverage, and inhibition efficiency for 304 SS corrosion in 1 M HCl in the absence and presence of MBT and MBT + KI at 25 °C.

Systems	Conc. (ppm)	WL (g)	${\varvec{C}}_{{\mathbf{R}}}$ (mm/Y)	S.D	ϴ	IE%
Blank 1M HCl	0.0	0.1460	3.3727	1.01 × 10^–2^	–	–
1M HCl + 0.1M KI	0.0	0.1189	2.7467	5.01 × 10^–2^	0.1856	18.56
MBT	100	0.0870	2.0098	1.11 × 10^–1^	0.4041	40.41
	200	0.0602	1.3906	1.03 × 10^–2^	0.5876	58.76
	400	0.0474	1.0950	1.53 × 10^–3^	0.6753	67.53
	600	0.0338	0.7808	3.51 × 10^–4^	0.7684	76.84
MBT + 0.1M KI	100	0.0190	0.4389	1.53 × 10^–4^	0.7901	79.01
	200	0.0131	0.3026	1.69 × 10^–2^	0.8555	85.55
	400	0.0110	0.2541	9.50 × 10^–4^	0.8784	87.84
	600	0.0074	0.1709	2.09 × 10^–3^	0.9179	91.79

**Figure 5:**
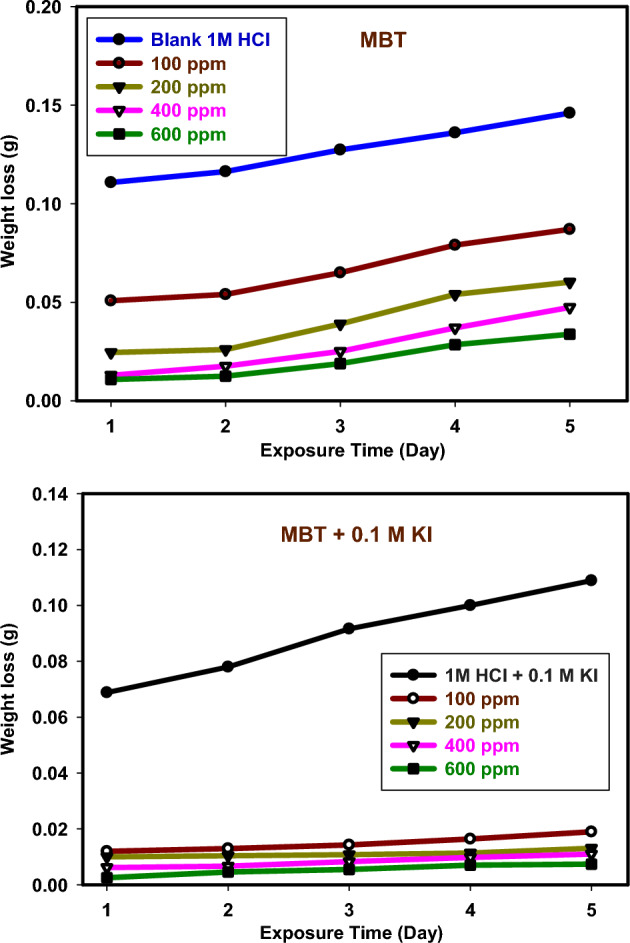
WL curve for 304 SS corrosion in 1 M HCl at various immersion times.

**Figure 6:**
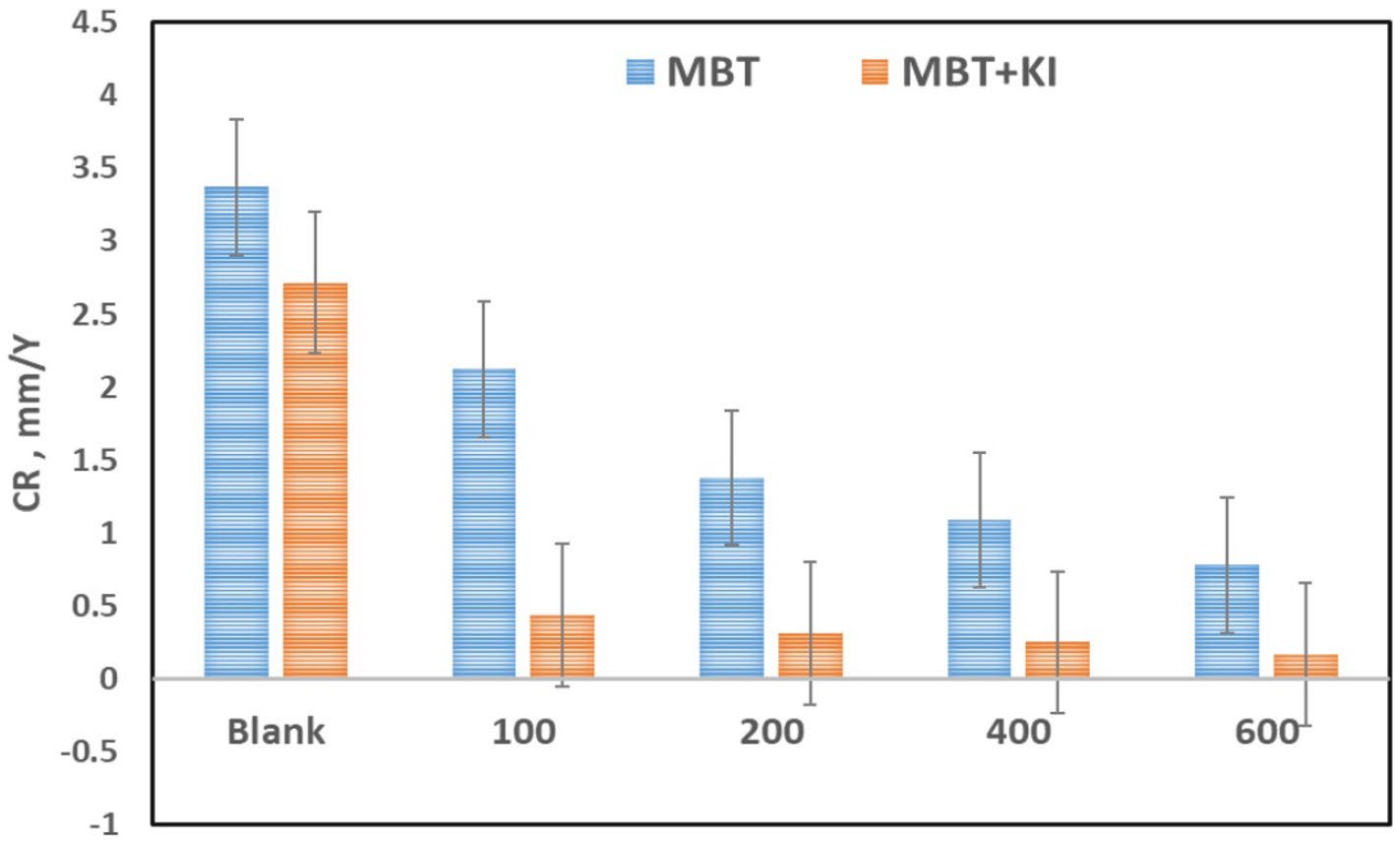
Error bar for comparing CR of 304 SS recorded in the system MBT and MBT + KI at different concentration.

Following the impact of concentration, this section will concentrate on immersion time because we are already aware of its influence on inhibitory performance. Studying its corrosion throughout a range of immersion times may be beneficial. Weight loss following varying immersion times in 1 M HCl with MBT and MBT + 0.1 M KI is shown in Fig. 5. These findings show that the IE % increases marginally throughout immersion time and peaks at 120 h, or 76.84% for the MBT inhibitor at an ideal concentration of 600 ppm. However, due to the significant reduction in the resistance of control, the efficiency of the examined compounds remains nearly consistent during a prolonged immersion time. The continued efficiency is a reflection of our chemical's capacity to create a solid, long-lasting protective layer on 304 SS surfaces surface in an acidic environment. The thickening of the film's formation is responsible for this outcome. According to earlier chemical research, the extent of dissolution is decreased^26,27^. Figure 6 displays the CR of 304 SS tracked in the MBT and MBT + KI systems at various concentrations. It can be demonstrated that the CR for systems MBT + KI is inhibitorier than without the addition of iodide, within the experimental error. We find that the synergistic efficiency of MBT inhibition has been substantially improved by the integration of KI.

#### PDP method

**Figure 7:**
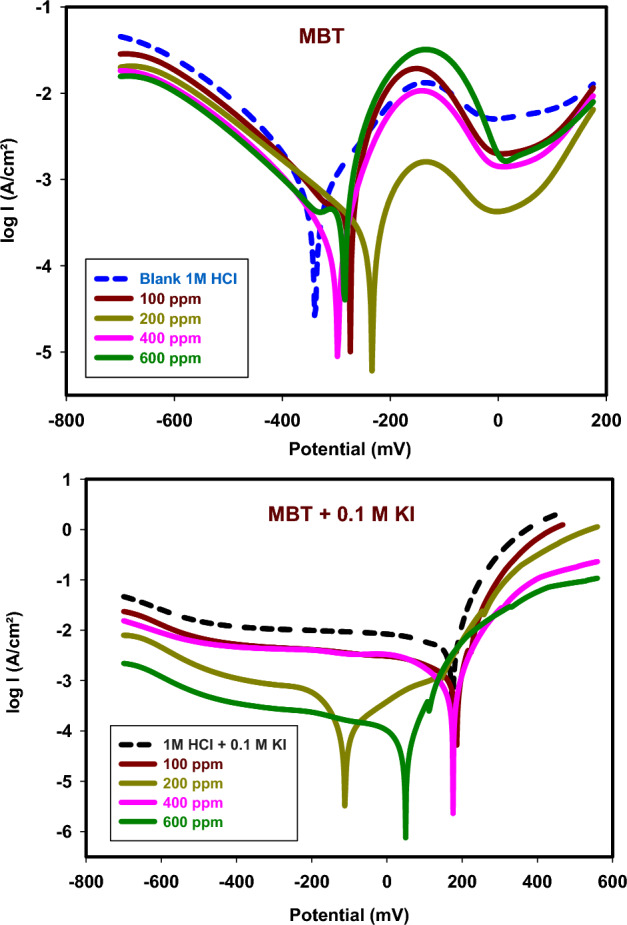
PDP curves for 304 SS in 1 M HCl in the absence and presence concentration of MBT and MBT + KI at 25 °C.

304 SS polarization curve in 1 M HCl with various concentrations of MBT with and without 0.1 M KI is depicted in Fig. 7. The potential corrosion (*E*_*corr*_), Tafel slopes (*β*_a_, *β*_c_), current density (*I*_*corr*_), rates of corrosion (CR), surface coverage (θ), S.D and efficiency (IE%) are presented in Table 2. The data demonstrate that adding concentrations of the Schiff base MBT reduces the values of *I*_*corr*_ and that this reduction is maintained when potassium iodide is also added. The adsorption of inhibitor molecules on the 304 SS by inhibiting the active sites on the steel surface is the cause of the decrease in *I*_*corr*_. The IE% readings in MBT only rise from 40.7 to 44.6% with increased concentration, but they grow from 62.3 to 90.6% in MBT with 0.1 M KI, indicating a greater surface coverage of the 304 SS. These results also demonstrate that MBT and KI work very well together to prevent corrosion of 304 SS. The anodic and cathodic reactions are therefore severely hindered. Although inhibitors can affect both cathodic and anodic processes, anodic reactions are primarily affected^12^. By adding more additives, the (*E*_*corr*_) shifted to more positive values, but the shift, which was less than 85 mV, indicated that the effects of MBT and MBT + 0.1 M KI were mixed type^28,29^.

**Table 2:** Corrosion parameters of PDP for 304 SS in 1M HCl in the absence and the presence concentration of MBT and MBT + KI at 25 °C.

Systems	Conc. (ppm)	*E*_*corr*_ mV	I_*corr*_ mA/cm^2^	*β*_*a*_ mV/dec	*β*_*c*_ mV/dec	R_p_ ohm.cm^2^	${C}_{{\text{R}}}$ (mm/Y)	S.D	ϴ	IE%
Blank 1 M HCl	0.0	− 344.9	0.3551	122.1	− 108.2	9.69	4.153	5.85 × 10^–3^	–	–
1M HCl + 0.1M KI	0.0	145.3	0.2816	28.1	− 136.3	23.62	3.294	1.40 × 10^–2^	0.206	20.6
MBT	100	− 282.2	0.2105	79.7	− 180.1	27.85	2.461	1.59 × 10^–2^	0.407	40.7
	200	− 240.4	0.2049	59.0	− 138.5	46.05	2.396	6.08 × 10^–3^	0.423	42.3
	400	− 304.6	0.2005	25.8	− 155.8	53.86	2.345	0.01 × 10^–3^	0.435	43.5
	600	− 293.4	0.1967	48.9	− 402.3	54.08	2.300	2.08 × 10^–2^	0.446	44.6
MBT + 0.1M KI	100	168.9	0.1339	91.8	− 412.9	87.19	1.567	7.00 × 10^–3^	0.623	62.3
	200	− 116.7	0.0924	51.7	− 334.3	92.31	1.081	1.00 × 10^–3^	0.739	73.9
	400	177.1	0.0647	192.7	− 102.0	211.80	0.756	1.00 × 10^–3^	0.818	81.8
	600	43.1	0.0335	50.8	− 55.2	325.71	0.391	1.53 × 10^–3^	0.906	90.6

#### EIS method

**Figure 8:**
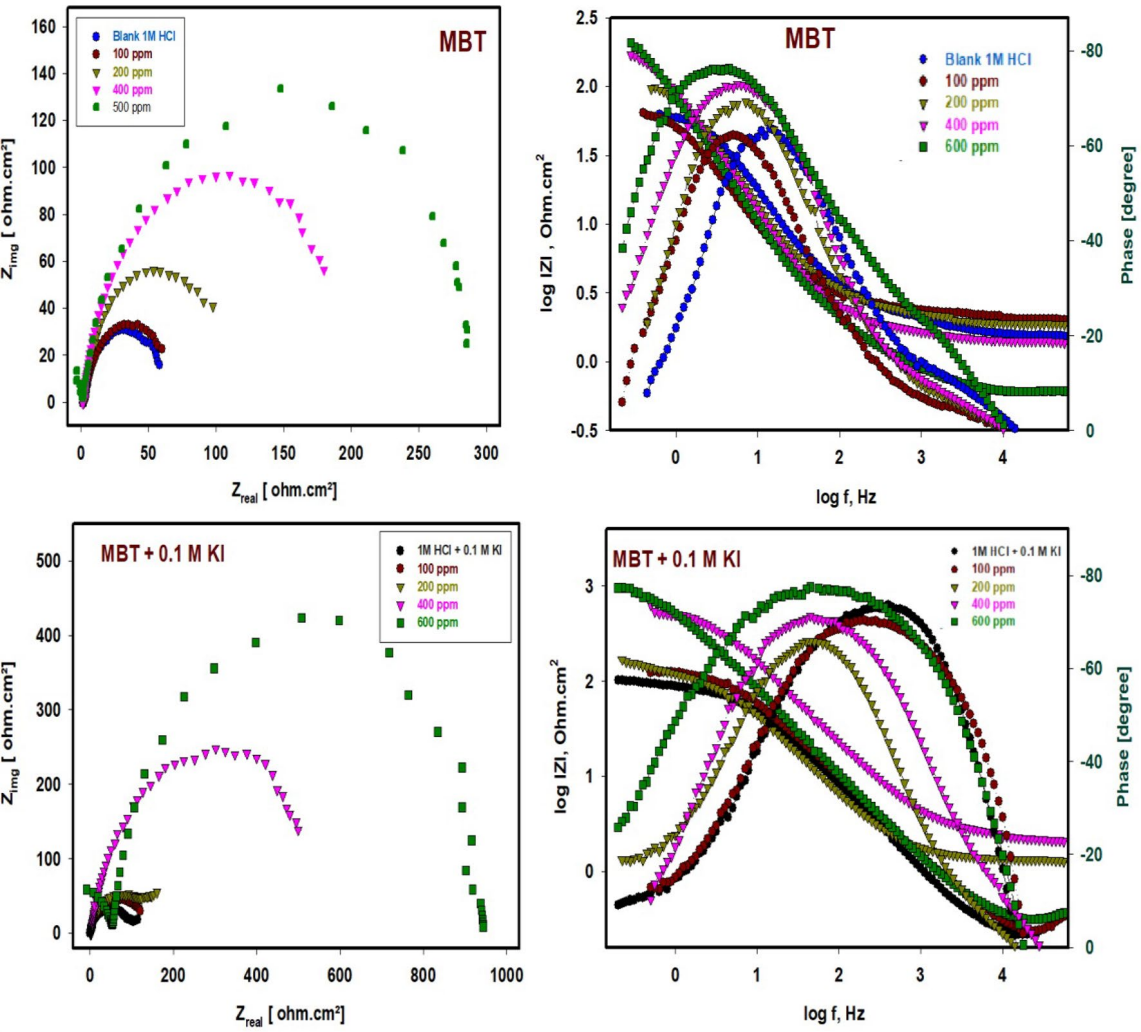
Nyquist and Bode plots for 304 SS in 1 M HCl in the absence and presence of concentration of MBT and MBT + KI at 25 °C.

Figure 8 display the Nyquist and Bode plots of 304 SS immersed for 30 min in 1 M HCl free and containing various concentrations of MBT both with and without 0.1 M KI, respectively. The impedance response of 304 SS specimens differs noticeably when the KI with MBT is present and when it is not. The impedance graphs resemble semi-circular shapes. The semicircle's depressed nature is a feature of solid electrodes that exhibit frequency dispersion. This phenomenon is linked to a number of physical phenomena, such as surface roughness and solid electrode inhomogeneities. The Bode modulus showed three regions: a low frequency zone that suggests charge transfer resistance, a middle frequency region that indicates capacitive resistance, and a high frequency region that indicates solution resistance.

**Figure 9:**
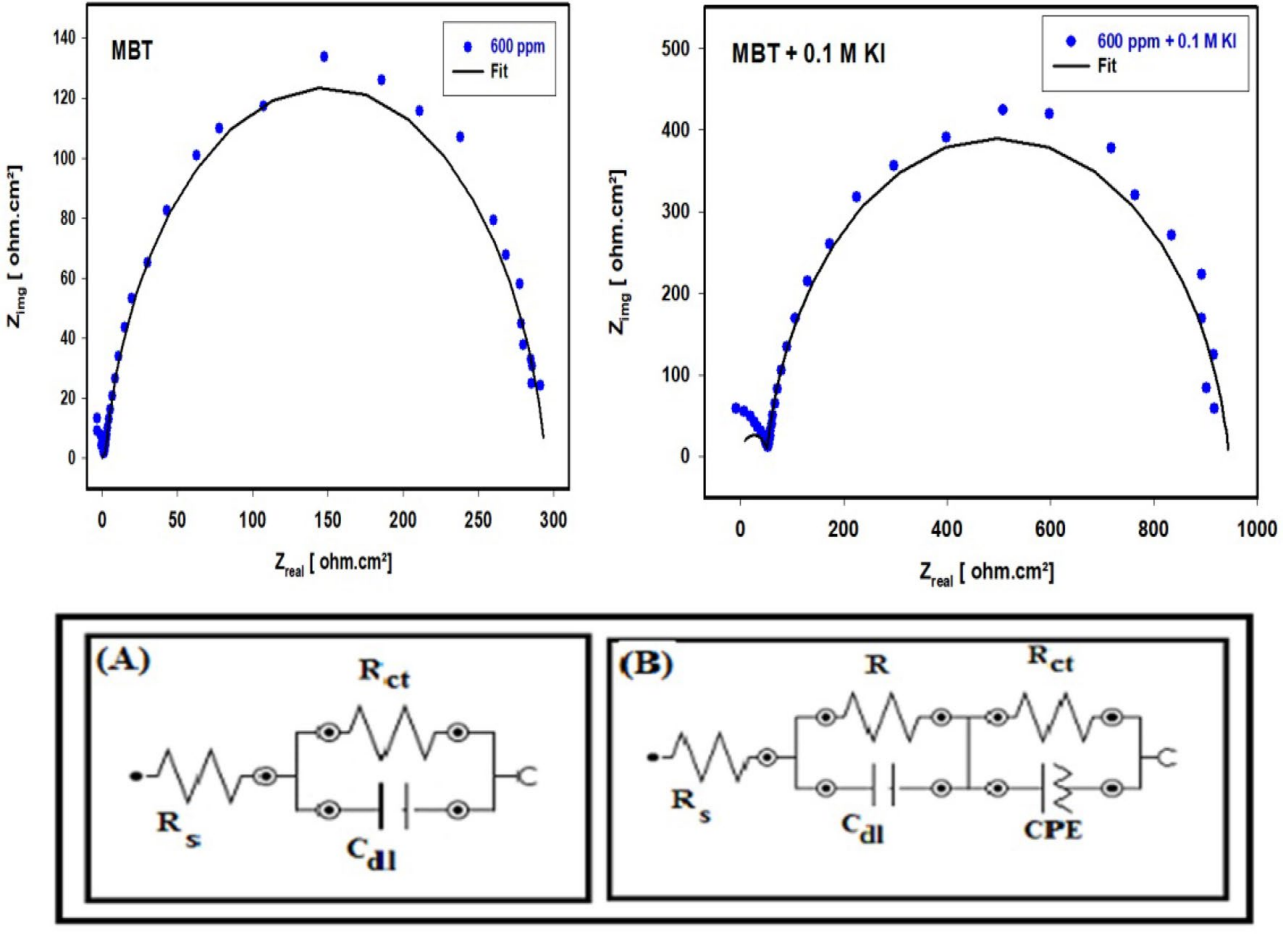
Comparison of fitted and investigational findings of 304 SS corrosion in the presence of MBT and MBT + KI and the equivalent circuit used (**A**, **B**).

When the concentration of the inhibitors increases, the absolute Bode modulus value shifts to higher values in the low frequency zone, once more suggesting the formation of a protective film and, thus, efficient corrosion inhibition by greater inhibitor concentrations than lower ones. In every scenario, the phase angles are approaching 80^0^. Pure electric models that might validate and make it possible under investigation can explain impedance behavior well^12^. Constant phase element (CPE) put into the circuit in place of C_dl_, which produces a more accurate illustrated in Fig. 9^30^. This reduces the effects caused by metal surface defects. Equation 7 can be used to express the impedance of CPE.

7 $$Z_{CPE} { } = \frac{1}{{Y_{0} \left( {j{\upomega }} \right)n}}$$

where Y_0_ is the magnitude of CPE, n is the phase shift, ω is the angular frequency, and j is the imaginary. CPE may be resistance, capacitance, and inductance depending upon the values of n^22^. EIS parameters and the efficiency of inhibition (IE%) are listed in Table 3. Values of CPE are reduced from 2.042 to 1.230 mFcm^−2^ in the case of MBT only but, in the presence of MBT with 0.1 M KI reduced from 2.042 mFcm^−2^ to reach 0.099 mFcm^−2^, indicating that MBT is adsorbing on the surface of 304 SS in 1 M HCl.

**Table 3:** Electrochemical impedance parameters for 304 SS in 1 M HCl in the absence and presence of a concentration of MBT and MBT + KI at 25 °C.

Systems	Conc. (ppm)	R_s_ Ω.cm^2^	R_ct_ Ω.cm^2^	CPE mF/cm^2^	n	R Ω.cm^2^	C_dl_ mF/cm^2^	*χ*^2^	Depletion angle °	IE%
Blank 1M HCl	0.0	1.32	43.99	2.042	0.811			2.5	− 1.23	–
1M HCl + 0.1M KI	0.0	1.40	113.40	0.538	0.723			3.1	− 5.99	61.28
MBT	100	1.38	92.70	1.586	0.601	0.58	0.935	5.2	− 1.58	52.55
	200	1.73	135.30	1.481	0.770	0.85	0.873	7.0	− 4.88	67.48
	400	1.94	200.60	1.287	0.813	1.26	0.769	6.5	− 10.4	78.07
	600	1.00	293.00	1.230	0.893	1.84	0.725	7.1	− 19.8	84.98
MBT + 0.1M KI	100	2.32	141.00	0.280	0.900	0.89	0.165	3.2	− 9.09	68.80
	200	5.93	186.60	0.278	0.819	1.17	0.164	5.3	− 10.7	76.43
	400	2.41	601.90	0.225	0.743	3.78	0.133	3.2	− 13.0	92.69
	600	5.12	973.78	0.099	0.914	8.50	0.004	2.8	− 13.6	95.48

The double layer that separates the charged metal surface from the solution and is thought of as an electrical capacitor may be used to explain this drop in C_dl_^31^. Values of R_ct_ and the width of the capacitive loop are increased by MBT concentrations in the absence and existence of KI. When utilized inhibitors are present, R_ct_ values rise from 43.99 to 293 Ω cm^2^ in the case of MBT alone, but rise from 43.99 to 973.78 Ω cm^2^ in the case of MBT plus 0.1 M KI, increasing the width of the capacitive loop and raising IE%. The IE% rises from 52.55 to 84.98% in MBT alone, but from 68.80 to 95.48% in MBT with 0.1 M KI. According to the IE percentage data, KI significantly improves MBT's ability to reduce the rate of corrosion. These findings imply that the MBT works via adsorption at the interface and that I ions strengthen this adsorption.

The importance of phase angle measurement during the application of electrical impedance spectroscopy was studied. The depletion or phase angle is shown in Table 3. Electrical impedance, phase angle (strength of capacitive character), and dissipation factor were scanned between 100 and 10,000 Hz current frequency. In low-frequency regions in which the frequency is less than 100 Hz, the phase angles of impedance in different stages are similar. When the frequency is higher than 100 Hz, there is a significant difference between the phase angles of different impedances^32,33^. The absolute value of the phase angle increases constantly with more negative values leading to more protection occurring. The negative phase angle equals the voltage lags the current. With an RL circuit, current lags voltage, so, when we divide the voltage by the impedance of an RL circuit, we want the resulting current to have a negative (lagging) phase.

The phase angles are then given by Eq. 8

8 $$\phi \, = {\text{ tan}}^{{ - {1}}} \left[ {\left( {{\text{X}}_{{\text{c}}} - {\text{ X}}_{{\text{L}}} } \right)/{\text{R}}} \right]$$

Note that if X_L_ = 0, meaning that there is no inductor in the circuit, then we arrive at the solution we obtained for the RC circuit. The enhancement in phase angle in the presence of MBT and MBT + KI molecules suggests that the adsorption of Schiff base compounds is what defines the capacitive routine of the surface of the metal/solution. More additives were adsorbed at the metal/electrolyte interface, generating an inhibitor-Fe complexing activity that was able to shield the metal surface from degradation and, as a result, increased a low corrosion rate. This is how the protective performance occurred^34^.

The comparison of fitted and investigational findings of 304 SS corrosion with MBT and MBT + KI is illustrated in Fig. 9. Utilizing the equivalent circuit example assigned in Fig. 9A, B. The ideal equivalent circuit was utilized to obtain the impedance characteristics to mathematically interpret the electrochemical performance^35^. The EEC illustrated in Fig. 9A was utilized to normalize the results of the inhibitor-free (blank) system, whereas Fig. 9B provides the Equivalent circuit with MBT and MBT + KI.

Nyquist plots of solutions inhibited by Schiff base MBT and MBT + KI were best represented by the equivalent circuit shown in Fig. 9B. In both cases (MBT and MBT + KI), according to the phase angle, two time constants are observed. Based on the discussion of the EIS spectra, the equivalent circuit of two-time constants shown in Fig. 9B was used for the modeling of the electrochemical processes of MBT and MBT + KI. R_s_ represents the electrolyte resistance, CPE-R_ct_ represents the resistive–capacitive response of the first time constant, and C_dl_-R represents the resistive–capacitive response of the second time constant. In the presence of MBT, from the Nyquist diagram, the apparent formation a time constant is observed between 1000 and 10,000 Hz whose capacitance decreases. From the Bode diagram in its impedance module format, in the high frequency region it is possible to observe the formation of the high frequency plateau starting at 1000 Hz; however, as time increases, its formation occurs at higher frequencies (> 10,000 Hz). In the region of intermediate frequency and low frequency, the apparent presence of a single linear relationship (log f–log |Z|) is observed, and it is not possible to define the low-frequency plateau. This suggests that the impedance modulus is greater than the recorded value.

In the presence of MBT + KI the maximum phase angles between 70° and 80° is very wide, suggesting at least two overlapping time constants. The first time constant shows an increase in its maximum phase angle and a displacement of its maximum at higher frequencies. The second time constant shows an increase in its maximum phase angle and a shift to lower frequencies. This behavior may be associated with the characteristics of the passive layer formed on MBT + KI; it is commonly accepted that the passive layer has a bilayer structure where the external part is porous (first time constant) and the internal part is compact and dense (second time constant) and therefore it presents the largest phase angle^32,33^. This result can reveal that there is more than one occurred electrochemical process, and the growth of more resistive MBT and MBT + KI layers adsorbed onto the SS surface, forming protected films.

The value of fitting was evaluated by chi error value (*χ*^2^) listed in Table 3. The values of the EIS fitting parameters as CPE, n, and R_ct_ are listed in Table 3. As seen from Table 3, the increase of inhibitor concentrations is ascribed to increasing the surface coverage by MBT and MBT + KI. It can be noticed that R_ct_ and R values increased with increasing concentrations. The dose-dependent increase in R_ct_ indicates a rise in inhibition efficiency across the board. Adsorption of the tested compounds is likely responsible for restoring the metal’s surface since the thickness of the adsorbed layer increases when inhibitor dosages are increased^36–38^. According to the above results, MBT and MBT + KI are suitable inhibitors for SS in HCl. Additionally, MBT in the presence of iodide exhibits a synergistic effect that enhances its inhibitory efficacy compared to MBT in the absence of iodide. There is a strong correlation between the findings from the polarization investigations and the impedance studies.

### Adsorption isotherm and thermodynamic variables

To analyze the data, several adsorption isotherms, including Langmuir, Temkin, and Freundlich, were fitted. Using data from EIS measurements, the surface coverage and inhibitory efficiency are estimated and displayed in Table 3.

#### Langmuir adsorption isotherm

**Figure 10:**
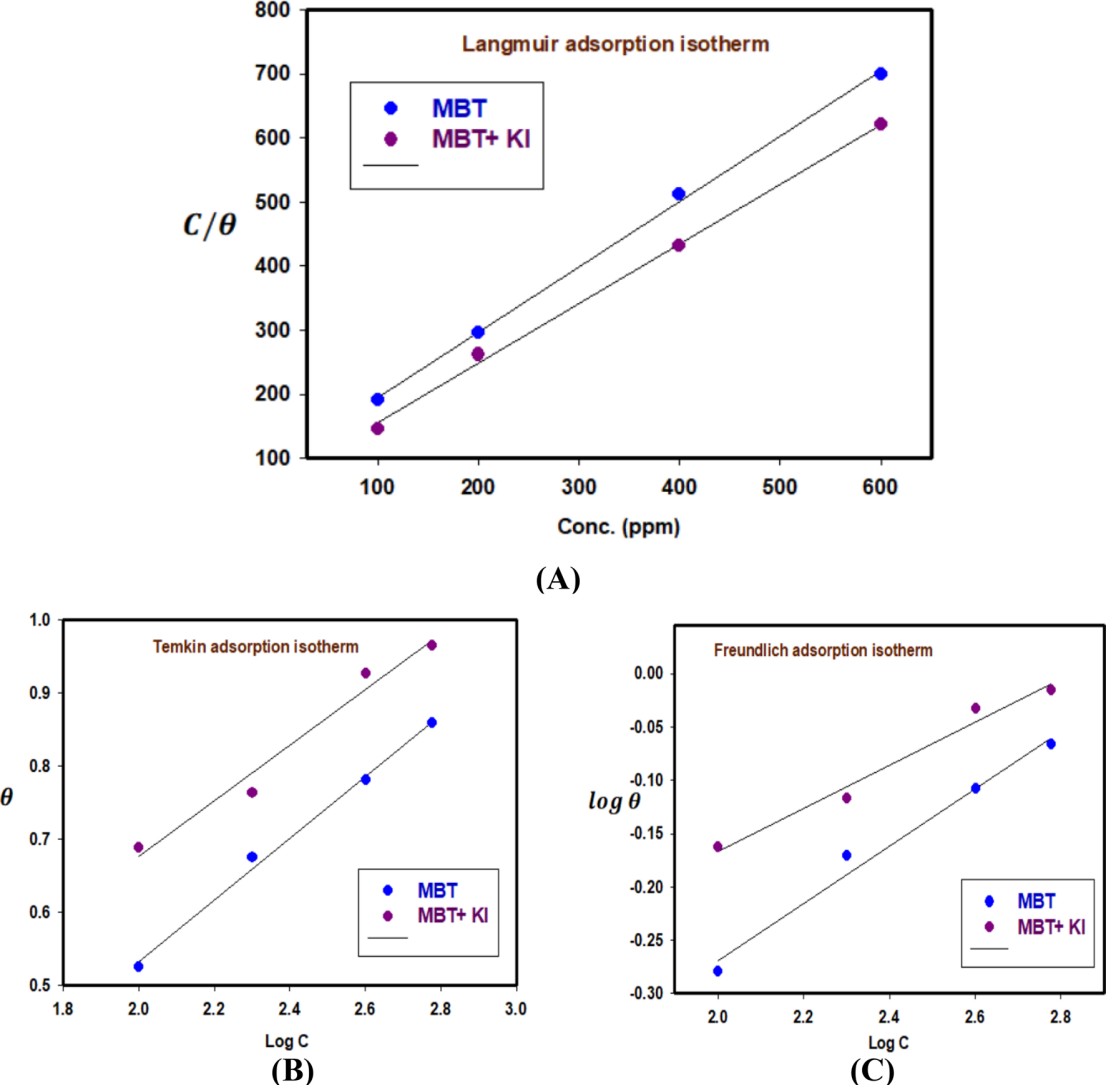
Adsorption isotherm plot from EIS for 304 SS in 1 M HCl in the absence and presence of concentrations of inhibitor at 298 K (MBT and MBT + KI).

To scrutinize the adsorption process of inhibitors, the surface coverage (θ) was graphically determined from the EIS method through a suitable fitting of adsorption isotherm. The best relation was obtained from the Langmuir adsorption isotherm through Eq. 9^16^.

9 $$\frac{{\text{C}}}{{\uptheta }} = {1}/{\text{K}}_{{{\text{ads}}}} + {\text{C}}$$

where C is inhibitor concentration and ${\text{K}}_{{{\text{ads}}}}$ is equilibrium constant. Figure 10A displays a plot C/${\uptheta }$ as the X-axis against C as the Y-axis, resembling the Langmuir adsorption isotherm. A perfect linear plot was produced with ${\text{R}}^{2}$ = 0.99827 and a slope of about 0.97796. It is customary that ΔG^0^_ads_ pertain to K_ads_. It could be computed by Eq. 10^11^:

10 $${\text{K}}_{{{\text{ads}}}} = { 1}/{55}.{\text{5 exp }}( - (\Delta {\text{G}}^{0}_{{{\text{ads}}}} /{\text{RT}})$$

where ∆${\text{G}}_{{{\text{ads}}}}$ is defined as the standard free energy of inhibitor adsorption, 55.5 is known as the molar concentration value of water in the solution, R is the gas constant, and T is the absolute temperature. Exploiting this previous equation, the ΔG^0^_ads_ of the inhibitor at 298 K was determined and tabulated in Table 4. When the values of ΔG^0^_ads_ are around − 20 kJ ${\text{mol}}^{ - 1}$ or lower, the adsorption is due to the electrostatic interaction between the charged molecules of the inhibitors and the charged electrode (physic-sorption). Meanwhile, those more negative than − 40 kJ ${\text{mol}}^{ - 1}$ reveal the charge transfer from the inhibitor to the surface of the metal to form a coordination bond (chemisorption)^21^. The type of observed adsorption (physisorption and chemisorption) is due to that the inhibitor includes more various chemical compounds that can be chemically adsorbed and the others can be physically adsorbed. In this current work, the obtained negative values of ΔG^0^_ads_ (less negative than − 20 kJ ${\text{mol}}^{ - 1}$) show that the adsorption process of the developed inhibitors on 304 SS in HCl solution is spontaneous and the adsorption technique of the studied inhibitors follows the physic-sorption.

**Table 4:** Adsorption parameters for 304 SS in 1 M HCl in the absence and presence of concentrations of inhibitor at 298 K (MBT and MBT + KI).

Isotherm	Investigated inhibitors	R^2^	Slope	K_ads_	∆G_ads_ kJ mol^−1^
Langmuir	MBT	0.99875	1.02160	0.01082	− 21.1644
	MBT + KI	0.99780	0.93432	0.01631	− 20.1486
Temkin	MBT	0.99488	0.42005	1.84645	− 7.02259
	MBT + KI	0.97412	0.37984	1.18077	− 3.78739
Freundlich	MBT	0.98276	0.26885	0.09331	− 9.21462
	MBT + KI	0.97836	0.20107	0.24528	− 8.55157

#### Temkin adsorption isotherm

According to Eq. 11^34^, the extent of surface covering is related to the inhibitor concentration and the adsorption equilibrium constant K_ads_.

11 $$\exp^{{\left( { - 2{\text{a}}\theta } \right)}} = K_{{{\text{ads}}}} \times C$$

where *a* is an attractive parameter and *K* is the adsorption constant. From Fig. 10B linear plots are obtained, which affirms that the adsorption obeys the Temkin adsorption isotherm. Adsorption parameters obtained from this Figure are shown in Table 4.

#### Freundlich adsorption isotherm

According to the Freundlich isotherm, *θ* is related to the inhibitor concentration *C* using Eq. 12^35^.

12 $${\text{log}}\theta = {\text{ log}}K_{{{\text{ads}}}} + n{\text{log}}C$$

where *n* is the empirical constant, and the other constants have the same meaning. Figure 10C shows straight lines relation of log *θ* against log *C* with slope *n* and intercept log *K*_ads_. The deduced adsorption parameters *K*_ads_, *n*, and Δ*G*^0^_ads_ are shown in Table 4. The adsorption process was studied using Langmuir, Freundlich, and Temkin isotherms. The adsorption studies indicated that the experimental data satisfied the Langmuir Freundlich and Temkin adsorption isotherms with good linearity. The chosen criteria of the best-fit isotherm are based on the higher correlation coefficient, *R*^2^. The higher value of K indicates that the inhibitor is strongly adsorbed on the 304 SS surface. From Table 4, and Fig. 10A, the fitting was obtained by Langmuir adsorption isotherm with high correlation coefficients (R) as well as with the other two models Temkin–Freundlich isotherm. As shown in Table 4, MBT and MBT + KI ΔG_ads_ values were negative (− 21.1644 and − 20.1486 kJ/mol, respectively), which indicates that their adsorption onto 304 SS surface was a spontaneous process that occurred through physic-sorption (physisorption). Many researchers apply adsorption isotherm to understand the mechanism of adsorption^36^. It is evident from the outcomes that linear R^2^ was ~ 0.99827, implying that the adsorption mechanism of MBT and MBT + KI molecules onto 304 SS surface obeyed Langmuir’s isotherm.

### Chemical kinetics of corrosion inhibiting

The integral method of analysis was used to examine the first-order kinetics. Equation 13^37^ gives this:

13 $$- \log \left( {\Delta W} \right){ } = \frac{{K_{1} t}}{2.303}$$

where ∆W is weight in (g), k_1_ is the rate constant in (Day^−1^), and t is the immersion in (Day). In addition, the t_0.5_ first-order reaction is given according to Eq. 14^3^:

14 $$t_{0.5} = \frac{0.693}{{K_{1} }}$$

**Figure 11:**
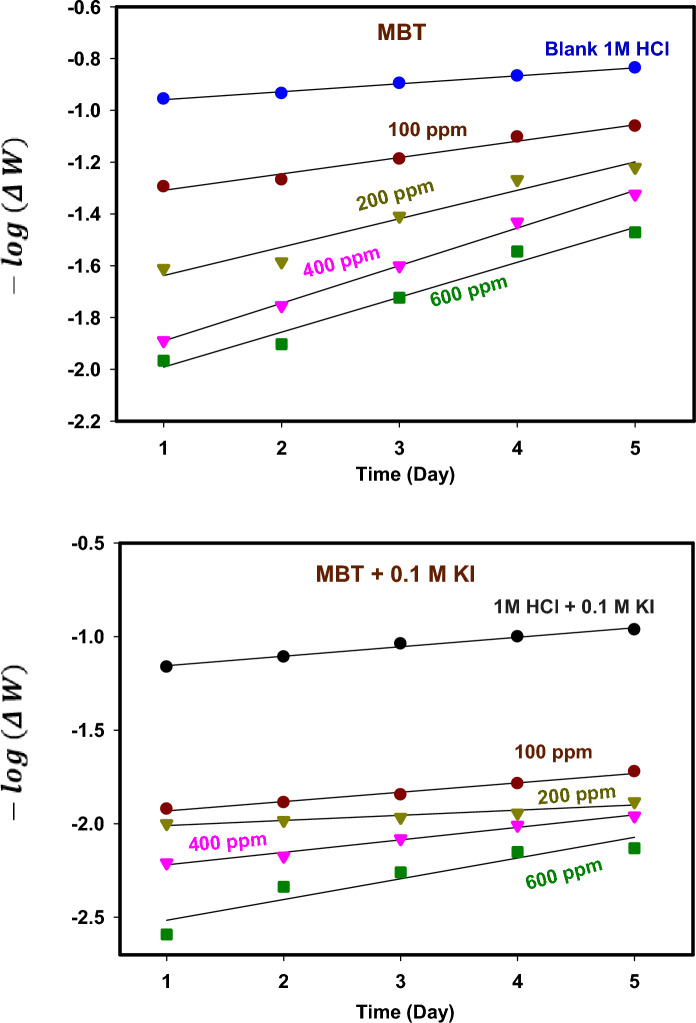
Chemical kinetic plot for 304 SS in 1 M HCl in MBT and MBT + KI at 298 K.

Figure 11 shows − log (∆W) against time in (Day) in the absence and presence of different concentrations of MBT and MBT + KI at 298 K. The rate constant and half-life parameters are tabulated in Table 5. The results revealed that the rate constants ($K_{1}$) of 304 SS in the presence of MBT or MBT + KI are higher than the rate constant of the blank solution (1 M HCl), while the half-life of 304 SS in the presence of MBT or MBT + KI were less than the half-life obtained for 1 M HCl solution. Based on the results of losing weight over time, the corrosion rates and half-life for 304 SS dissolution were somewhat inhibited. These MBTs are thought to be effective inhibitors because they can reduce the amount of time it takes for metals to become corrosion products^38^. The high value of R^2^ fitted well with the first-order kinetics conformity with reports of Ijuo et al.^39^.

**Table 5:** Chemical kinetic parameters for 304 SS in 1 M HCl in MBT and MBT + KI at 298 K.

Systems	Conc. (ppm)	${K}_{1}$ Day^−1^	${t}_{0.5}$ Day	R^2^
Blank 1M HCl	0.0	0.07082	9.7854	0.99409
1M HCl + 0.1M KI	0.0	0.11674	5.9363	0.98358
MBT	100	0.14567	4.7573	0.97503
	200	0.25286	2.7406	0.95108
	400	0.33464	2.0708	0.99608
	600	0.31065	2.2308	0.97384
MBT + 0.1M KI	100	0.11515	6.0182	0.98321
	200	0.16319	4.2465	0.90664
	400	0.15368	4.5094	0.98418
	600	0.25532	2.7142	0.88471

### Synergistic impact of KI on effectiveness inhibition

Because halide ions have a stronger propensity to be adsorbed on the surface in interaction with cations by chemisorption^26^, it is possible to explain the synergistic inhibition by the combination of MBT and I^−^ for corrosion 304 SS in 1 M HCl. Inhibition synergism is made by increased surface coverage by electrostatic interaction with protonated Schiff base. Steel anodic and cathodic reactions are polarized by I^−^ throughout a broad potential range. It becomes clear that covalent bonding to the metal must be involved and that the effects of I^−^ are not solely due to electrostatic forces. Electron pair bonding is rendered feasible by the huge size and simple polarizability of I^−^. Adsorption of the halide ions on the metal surface is followed by the inhibitor being pulled into the double layer by the halide ion, resulting in the formation of ion pairs right on the surface^12^. The synergy between MBT and negatively charged I is interpreted as the cause of the efficiency increase. Studying the key component of the synergistic impact (S) parameter is required to further explore the contribution of I^−^ to the enhancement of the adsorption mechanism. The following Eq. 15^40^ is used to compute the synergism parameter (SI).

15 $$S_{{\text{I}}} = \frac{{1 - {\text{I}}_{1 + 2} }}{{1 - {\text{I}}^{\prime}_{1,2} }}$$

Herein, (I_1+2_) = I_1_ + I_2_; I_1_ = efficiency of halide ions; I_2_ = efficiency of used inhibitors, and (I′_1, 2_) = efficiency for a mixture of used inhibitors and iodide ions. S values larger than one indicate a synergistic impact whilst, S values less than one indicate an antagonistic action that may result in competing adsorption. Applying the final equation to the efficacies of the experimental methods utilized, such as the gravimetric method, PDP, and EIS technique, this factor was evaluated. The obtained synergism parameter is listed in Table 6. In contrast to systems inhibited using MBT alone, EIS data demonstrate that MBT + KI systems provided the highest value of inhibition efficacy. The interaction between KI and MBT inhibitors has been confirmed by a synergy parameter derived from the EIS data. It is believed that the stability of negative iodide ions and MBT enhances the surface area covered and, as a result, the efficiency value. This demonstrates the synergistic nature of the improved inhibitory efficacy brought about by the addition of iodide ions to MBT^41^.

**Table 6:** Synergism parameter (S_I_) for concentrations of MBT on 304 SS in combination with 0.1 M KI.

Conc. (ppm)	S_I_ (Wt Loss method)	S_I_ (PDP method)	S_I_ (EIS method)
100	0.7431	0.8041	1.6642
200	0.9026	1.0875	1.6937
400	0.9798	1.4802	1.5088
600	1.0397	2.7247	1.5294

### Theoretical studies of MBT inhibitor

**Figure 12:**
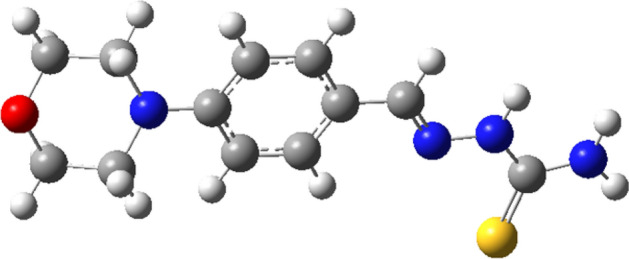
Optimized structure of inhibitor (MBT).

**Figure 13:**
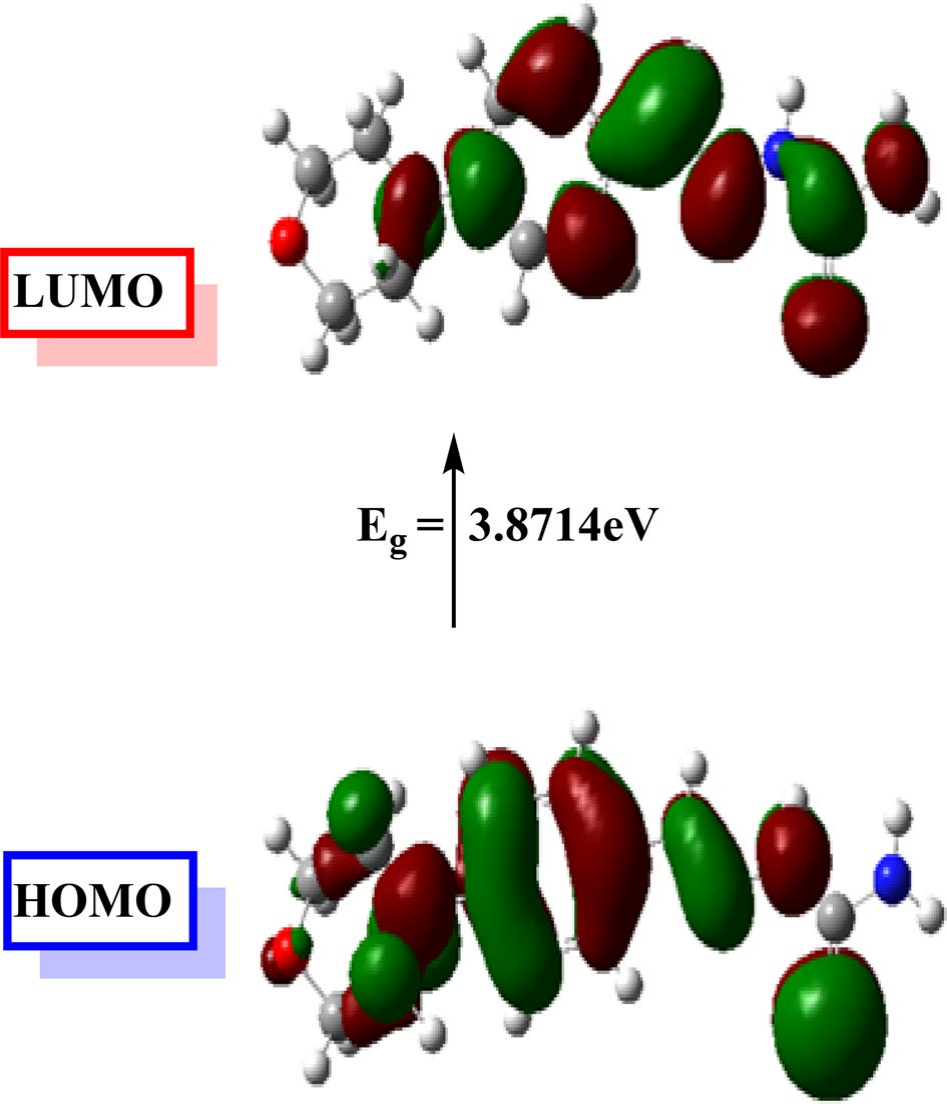
MO and their energies for MBT inhibitor.

The examined inhibitor's optimized molecular structure, together with the associated highest occupied frontier molecular orbital (HOMO), and lowest unoccupied frontier molecular orbital (LUMO), are given in (Figs. 12, 13). The HOMO and LUMO energies are correlated with percent inhibition efficiencies. The percent inhibition efficiencies increase if the molecules have higher HOMO energies and lower LUMO energies^24^. The percent inhibition efficiency increased with a decrease in energy gap (ΔE). The values for E_HOMO_, E_LUMO_, and ΔE show that MBH has a somewhat greater ability to act as a corrosion inhibitor. To gain a better understanding of how inhibitor molecules interact with metal surfaces, the present study discusses several additional parameters, including global hardness (η), global softness (σ), electronegativity (*X*), chemical potential (u), global electrophilicity (ω), fraction of electron transfer (ΔN) and energy associated with a backing donation (E_b−d_), Table 7. Furthermore, the relationships used in all of these calculations are presented previously in our publications^24,42^. In general, an inhibitor with a lower global hardness value and a greater softness value corresponds to high chemical reactivity and demonstrates high inhibitory efficiency^25^. Additionally, all of the Schiff base compounds' chemical potential values are negative, as shown in Table 7, indicating that the compounds are stable. Additionally, the MBT inhibitor's high potential value (− 3.4377 eV) and low electrophilicity value (3.4377) favor its nucleophilic behavior^43^, this outcome is consistent with the HOMO and LUMO energy values^44^. Fraction of electron transfer (ΔN), this quantity is commonly used to describe a molecule's ability to accept or transfer electrons to or from a metal^44^. If ΔN is more than zero, the inhibitor can donate its electron to the metal, and if ΔN is less than zero, the opposite happens^45^. Thus, the positive values of ΔN for the inhibitor studied imply that the electron donation is from the inhibitor to the metal surface. The negative sign of E_b−d_ suggests that metal-to-inhibitor back donation is energetically beneficial. Back donation and donation methods improve inhibitor adsorption on the iron surface. These findings correspond with the experimental inhibition efficiency.

**Table 7:** Quantum chemical parameters calculated for CMBAH inhibitor.

Parameters	MBH
Total energy, (Hartree)	− 1159.2506
Dipole moment, (Debye)	6.5671
Chemical potential u, ( eV)	− 3.4377
Electronegativity *X*, ( eV)	3.4377
E_HOMO_, (eV)	− 5.3734
E_LUMO_, ( eV)	− 1.5020
ΔE, (eV)	3.8714
η, ( eV)	1.9357
σ, (eV^−1^)	0.5166
∆N	0.9201
ω	3.0525
E_b−d_ (eV)	− 0.4839

#### Molecular electrostatic potential (MEP)

**Figure 14:**
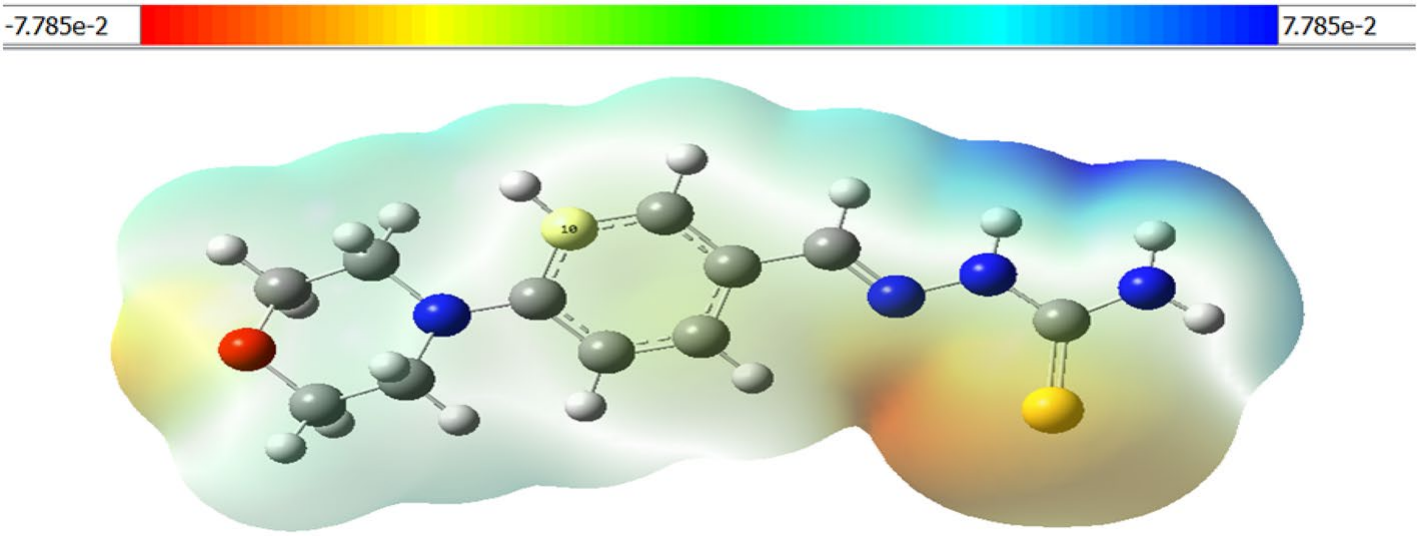
Molecular electrostatic potential (MEP) surface and counterplots for MBT inhibitor.

The molecular electrostatic potential is used to predict the reactivity of inhibitor molecules and the overall charge distribution^46^. As can be seen in Fig. 14**,** the B3LYP with the basis set 6-31G optimized results were used to create the MEP map of the compound MBT. Colors are used to denote the various MEP surface zones. Indeed, the zone from red to green is favorable for electrophilic attacks. While the zone from green to blue can be attacked by nucleophiles, the green zone represents zero potential.

### Biological activity

The newly synthesized Schiff base ligand (MBT) were investigated for their inhibitory effects on the growth of *Bacillus cereus* (G +ve), *E. coli* (G −ve), *Micrococcus luteus* (G +ve), *Pseudomonas aeruginosa* (G −ve), *Serratia marcescens* (G-ve), *Staphylococcus aureus* (G +ve) bacteria and *Aspergillus flavus*, *candida albicans*, *Fusarium oxysporum*, *Geotrichum candidum*, *Scopulariopsis brevicaulis* and *Trichophyton rubrum* fungi. The antimicrobial activity was tested by using the disc diffusion method; the clear zone of inhibition around each disk was measured (in mm) and compared to the known sensitive drugs: chloramphenicol (CHL) as an antibacterial drug and clotrimazole (CLO) as an antifungal drug. The antibacterial and antifungal activities of the prepared MBT are reported in Table 8. The findings suggest that the MBT exhibits greater activity against *Aspergillus flavus*, while the MBT exhibits no activity against the tested bacteria under the experimental conditions.

**Table 8:** Antibacterial and antifungal activity (inhibition zone in mm) of chemical compounds.

Bacteria	Compound	
	MBT	CHL
*Bacillus cereus* (G +ve)	0	22
*E. coli* (G −ve)	0	20
*Micrococcus luteus* (G +ve)	0	20
*Pseudomonas aeruginosa* (G −ve)	0	18
*Serratia marcescens* (G −ve)	0	20
*Staphylococcus aureus *(G + ve)	0	18

Fungi	Compound	
	MBT	CLO
*Aspergillus flavus*	16	24
*Candida albicans*	0	22
*Fusarium oxysporum*	0	18
*Geotrichum candidum*	0	28
*Scopulariopsis brevicaulis*	13	22
*Trichophyton rubrum*	14	42

### Corrosion inhibition mechanism

It is necessary to provide an adsorption mechanism to clarify the mechanism of inhibition of the examined molecules during the creation of a protective layer on the metal surface. The inhibitory molecules adsorb on the metal surface due to their strong electrical characteristics, according to the primary findings of the studies and theoretical approaches. Due to chemical and physical adsorption, the generated layers' protective performance has also been demonstrated. It is typically believed that the corrosion process of steel can take place in the unhindered HCl solution in the following ways:

16 $${\text{Fe }} + {\text{ Cl}}^{ - } \leftrightarrow \, \left( {{\text{FeCl}}} \right)_{{{\text{ads}}}} + {\text{ e}}^{ - }$$

17 $$\left( {{\text{FeCl}}} \right)_{{{\text{ads}}}} \leftrightarrow \left( {{\text{FeCl}}^{ + } } \right) + {\text{ e}}^{ - }$$

18 $$\left( {{\text{FeCl}}^{ + } } \right) \leftrightarrow {\text{Fe}}^{{{2} + }} + {\text{Cl}}^{ - }$$

It observed that the dissolution of the steel in acid has positively charged. The inhibitor compounds typically become protonated when they are present in an acidic environment. In our situation, the protonated form of MBT is expressed as follows when considering the adsorption process:

19 $${\text{MBT }} + {\text{ H}}^{ + } \leftrightarrow \, \left[ {{\text{MBT }} + {\text{ H}}} \right]^{ + }$$

**Figure 15:**
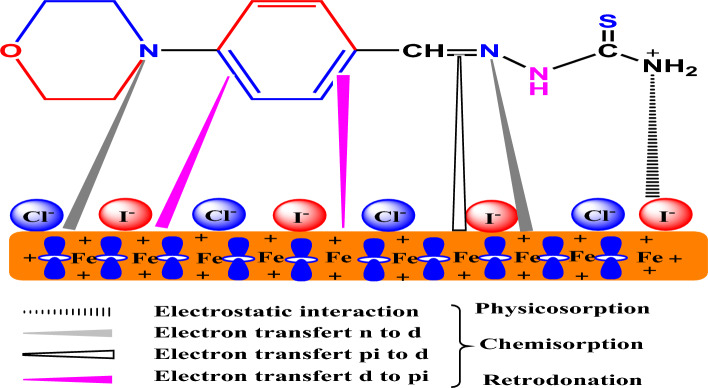
Inhibition mechanism of MBT adsorption on 304 SS.

Due to I^−^ large ionic radius and high hydrophobicity, they participated in the adsorption process onto the steel surface, through a synergetic effect that caused corrosion inhibition. Then under the synergistic effect of MBT + KI, a self-assembled [Fe(0) Fe(II) I^-^MBT] film can be formed (Fig. 15). The enhanced efficacy of inhibitors in the presence of I^−^ is thus explained by a greater likelihood of donor–acceptor interactions. MBT seems to be a great inhibitor in 1 M HCl, and its potential was enhanced by KI since it achieved 96.57%.

## Conclusion

The research summary is based on several significant discoveries, including the following, which are all based on the outcomes of the experiments used:

- MBT is a mixed-type inhibitor according to potentiodynamic polarization data because it prevents the corrosion of 304 SS in acid solution.
- The inhibition efficiency increases with an increase in the concentration of inhibitor.
- The interpretation of the improvement in efficiency is attributed to the synergy between MBT and negatively charged iodide ions. A cooperative effect of iodide ions with MBT is an occurrence.
- The addition of iodide ions to MBT enhanced the inhibition efficiency due to the synergistic effect.
- Adsorption of MBT alone and in mixing with iodide ions on the surface of steel were found to strongly fit Langmuir's adsorption isotherm.
- The thermodynamic parameters calculated from the adsorption isotherms showed that physisorption is involved in the inhibition process.
- DFT findings correspond with the experimental inhibition efficiency.
- MBT exhibits greater activity against *Aspergillus flavus*, while the MBT exhibits no activity against the tested bacteria under the experimental conditions.
- Furthermore, quantum chemical calculations were employed to correlate the experimentally determined inhibitory efficiency.

### Supplementary Information

Supplementary Information.

## Data Availability

All data generated or analyzed during this study are included in this published article and its supplementary information files.
