# Supplementary material for: Synthesis, characterization, synergistic inhibition, and biological evaluation of novel Schiff base on 304 stainless steel in acid solution
Source: Sci Rep. 2024 Jan 4;14:470. doi: 10.1038/s41598-023-51044-w (PMC10764748; doi:10.1038/s41598-023-51044-w)
Supplement: Supplementary file 1 — Supplementary Information. [file 41598_2023_51044_MOESM1_ESM.docx]

**Table S1. Analytical data and some physicochemical properties of MBT**

| **Compound** | **Chemical**  **Formula** | **Color** | **m.p.**  **(^o^C)** | **%Yield** | **M.Wt**  **(gm/mol)** | **Found % (calcd %)** | | | | **Ω_m_**  **(ohm^-1^**  **cm^2^mol^-1^)** |
| --- | --- | --- | --- | --- | --- | --- | --- | --- | --- | --- |
|  |  |  |  |  |  | % C | % H | % N | % S |  |
| MBT | C_12_H_16_N_4_OS | Yellow | 270 | 62 | 264.34 | 54.47 (54.52) | 6.05 (6.10) | 21.18 (21.19) | 12.10 (12.13) | - |

**
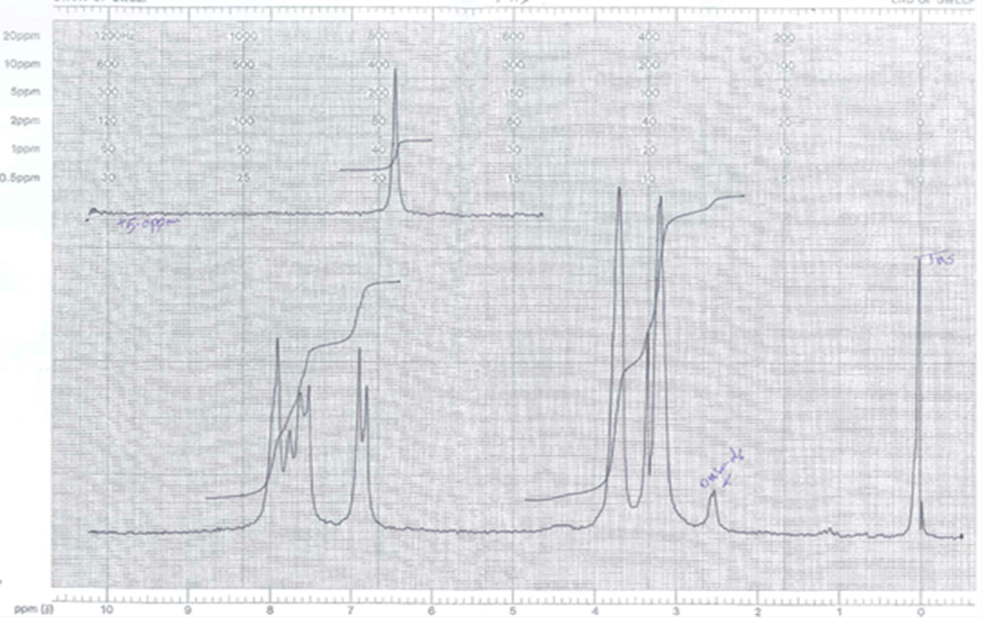
**

**Figure S1. ^1^H NMR spectrum of MBT.**

**
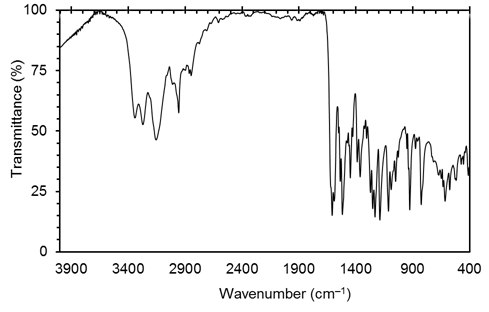
**

**Figure S2. FT-IR spectra of MBT.**

**
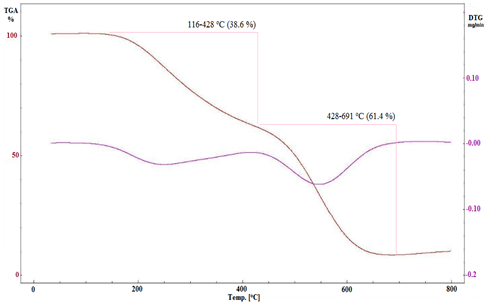
**

**Figure S3. TG-DTG curves of MBT .**

**Figure S4. Thermal decomposition of MBT.**
